# Legislation of direct-to-consumer genetic testing in Europe: a fragmented regulatory landscape

**DOI:** 10.1007/s12687-017-0344-2

**Published:** 2017-11-18

**Authors:** L. Kalokairinou, H. C. Howard, S. Slokenberga, E. Fisher, M. Flatscher-Thöni, M. Hartlev, R. van Hellemondt, J. Juškevičius, J. Kapelenska-Pregowska, P. Kováč, L. Lovrečić, H. Nys, A. de Paor, A. Phillips, L. Prudil, E. Rial-Sebbag, C. M. Romeo Casabona, J. Sándor, A. Schuster, S. Soini, K. H. Søvig, D. Stoffel, T. Titma, T. Trokanas, P. Borry

**Affiliations:** 1 Department of Public Health and Primary Care, Centre for Biomedical Law and Ethics, University of Leuven, Kapucijnenvoer 35, Box 7001, 3000 Leuven, Belgium; 2 Centre for Research Ethics and Bioethics, Uppsala University, Uppsala, Sweden; 3 Faculty of Law, Uppsala University, Uppsala, Sweden; 4 Robert Koch-Institute, Berlin, Germany; 5 Institute of Public Health, Medical Decision Making and Health Technology Assessment, UMIT - University for Health Sciences, Medical Informatics and Technology, Hall in Tirol, Austria; 6 Faculty of Law, University of Copenhagen, Copenhagen, Denmark; 7 Leiden University Medical Centre, Leiden, the Netherlands; 8 Faculty of Law, Mykolas Romeris University, Vilnius, Lithuania; 9 Faculty of Law and Administration, Nicolaus Copernicus University, Toruń, Poland; 10 Forensic.sk Inštitút forenzných medicínskych expertíz s.r.o, Bratislava, Slovakia; 11 Department of Criminal Law and Criminology, Faculty of Law, Trnava University, Trnava, Slovakia; 12 Clinical Institute of Medical Genetics, University Medical Centre Ljubljana, Ljubljana, Slovenia; 13 School of Law and Government, Dublin City University, Dublin, Ireland; 14 School of Law, Trinity College Dublin, University of Dublin, Dublin, Ireland; 15 AK PRUDIL a spol, Brno, Czech Republic; 16 Institut national de la santé et de la recherche médicale, Paris, France; 17 The University of the Basque Country, Leioa, Spain; 18 Faculty of Political Science, Legal Studies and Gender Studies of the Central European University, Budapest, Hungary; 19 Department of Legal Sciences, University of Trento, Trento, Italy; 20 Helsinki University Central Hospital, Helsinki, Finland; 21 Faculty of law, University of Bergen, Bergen, Norway; 22 University Paris-Est Créteil, Créteil, France; 23 School of Governance, Law and Society, Tallinn University, Tallinn, Estonia; 24 School of Information Technologies, Tallinn University of Technology, Tallinn, Estonia; 25 School of Law, European University of Cyprus, Egkomi, Cyprus

**Keywords:** Direct-to-consumer genetic tests, Regulation, In vitro diagnostic medical devices, Medical supervision, Genetic counselling, Informed consent

## Abstract

Despite the increasing availability of direct-to-consumer (DTC) genetic testing, it is currently unclear how such services are regulated in Europe, due to the lack of EU or national legislation specifically addressing this issue. In this article, we provide an overview of laws that could potentially impact the regulation of DTC genetic testing in 26 European countries, namely Austria, Belgium, Cyprus, the Czech Republic, Denmark, Estonia, Finland, France, Germany, Greece, Hungary, Ireland, Italy, Latvia, Lithuania, Luxembourg, Norway, Poland, Portugal, Romania, Slovakia, Slovenia, Spain, Sweden, the Netherlands and the United Kingdom. Emphasis is placed on provisions relating to medical supervision, genetic counselling and informed consent. Our results indicate that currently there is a wide spectrum of laws regarding genetic testing in Europe. There are countries (e.g. France and Germany) which essentially ban DTC genetic testing, while in others (e.g. Luxembourg and Poland) DTC genetic testing may only be restricted by general laws, usually regarding health care services and patients’ rights.

## Introduction

Since the 1960s, genetic tests have been provided to patients for health-related reasons within a clinical setting (Sanfilippo et al. 2015), usually following a medical referral, genetic counselling, and upon obtaining informed consent. However, in the last decade, a wide variety of direct-to-consumer (DTC) genetic tests have also been made available by commercial companies with varying involvement of healthcare professionals. In some cases, genetic tests are advertised DTC, but then ordered by a healthcare provider, who may also be the recipient of the test results; in other cases, tests are advertised, sold and delivered DTC without any involvement of a medical professional (Hogarth et al. 2008). In the latter case, the consumer usually orders a genetic test online. Subsequently, he/she receives a testing kit at home to collect a biological sample (which, depending on the test, usually comprises of an accumulated volume of saliva or hair). After sending the biological sample to the company, genetic material is extracted from it, and the DNA is analysed. A few weeks later, the consumer is provided with the test results, which are sent to him/her either via email or upon accessing a secure website (Kalokairinou et al. 2014).

Currently, a large number of both health-related and non-health-related genetic tests is available directly to consumers (Phillips 2016). The former group includes diagnostic tests for monogenic diseases such as cystic fibrosis, susceptibility tests for common complex disorders such as cardiovascular disease and diabetes, carrier tests for X-linked and recessive disorders, as well as pharmacogenomic and nutrigenomic tests. Non-health-related tests include ancestry tests, athletic performance tests, matchmaking tests and tests for ‘fun’ traits, such as ear wax type and eye colour (EASAC and FEAM Working Group 2012).

This model of providing genetic testing has been very controversial among stakeholders over the past years. On the one hand, proponents have been underlining that DTC genetic testing enhances the autonomy of consumers (Vayena 2015) and allows them to be in charge of their healthcare management (Marietta and Mcguire 2009), all without the intermediary of doctors and long waiting lists for hospital appointments. Furthermore, it has been argued that consumers have a right to their genetic information (Su et al. 2013) and that, by circumventing the public healthcare system, the privacy of genetic data may be better protected against insurers and employers.

On the other hand, this model of providing genetic tests has also been subject to criticism. The absence of medical supervision and genetic counselling often encountered in the commercial setting has raised concerns regarding potential misinterpretation of test results by consumers, which may lead to unnecessary distress and/or inappropriate healthcare decision making (Howard and Borry 2012). Moreover, it has been claimed that information accompanying the tests are often misleading or inadequate, reducing informed consent to merely ticking a box (Hogarth et al. 2008). Further concerns focus on the unproven or unclear clinical validity and utility of many of the tests offered DTC (Bunnik et al. 2011; European Society of Human Genetics 2010) the potential inappropriate testing of minors (Howard et al. 2011), surreptitious testing of third parties (Tamir 2010), endangering the privacy of genetic data, especially in cases of company bankruptcy or change of ownership (Zawati et al. 2011) and downstream costs to the healthcare system from inappropriate follow-up visits to healthcare professionals (Giovanni et al. 2010). This argument is mostly prevalent in countries with a national tax based funded healthcare system.

Of particular importance is that the rapid advance of genotyping technologies and their decreasing costs have made DTC tests increasingly available to consumers, outpacing the development of effective regulation of such commercial services (Grimaldi et al. 2011). Especially with regard to Europe, currently, to the best of our knowledge, there is no EU or national legislative instrument, specifically regulating DTC genetic testing. As a result, different aspects of genetic testing may be regulated by separate and sometimes overlapping legal instruments on the national, EU or international level.

Laws affecting the regulation of DTC genetic testing on the EU level include consumers’ protection laws and more specific laws on in vitro diagnostic (IVD) medical devices. For example, the Unfair Commercial Practices Directive 2005/29 EC (European Parliament and the Council of the EU 2005) aims to protect consumers from misleading actions and omissions, as well as from aggressive commercial practices coming from traders, such as emotional pressure or deceptive advertising (European Commission 2016). In addition, genetic tests with a medical purpose are considered IVD medical devices and, in addition to being subject to consumer protection laws, their safety and efficiency are also regulated by stricter laws compared to common commercial goods. More specifically, the Directive 98/79 EC on In Vitro Diagnostic Medical Devices (European Parliament and the Council of the EU 1998) aims to ensure that all such devices entering the European market are safe and efficient. This Directive (which will be replaced by a new Regulation in May 2022) deals, among others, with issues of analytical and, arguably, clinical validity of genetic tests, as well as labelling.

Moreover, there are aspects of genetic testing that go beyond the regulation of genetic tests as products and into the realm of services, such as the provision of appropriate medical supervision and genetic counselling, including the informed consent process. While the existing EU regulatory framework covers, to a large extent, genetic tests as products, the regulation of aspects of genetic testing akin to services is not granted the same level of control (Nicol et al. 2016). The provision of genetic tests in Europe has traditionally been seen as directly linked to the healthcare services and has been regulated mostly on the national level (Godard et al. 2003). Many European countries restrict the framework within which genetic tests are provided to patients by regulating the type of tests that may be offered to the public, the type of facilities where genetic tests may take place and the qualifications of the personnel offering such testing.

To this end, some countries implement biomedical and/or bioethical regulation [such as Norway (Bioteknologiloven 2003), Spain (Boletín Oficial del Estado No159 2007) and France (LOI n° 2011–814 2011)^1^)]; others provide laws specific to genetics (Soini 2012), [such as Austria (Gentechnikgesetz 1994), Germany (Gendiagnostikgesetz 2009),^2^ Hungary (Genetikai törvény 2008) and Sweden (Lag (2006:351) 2006, Chapter 1, Section 1)] or address genetics within more general laws on issues related to healthcare [such as the Czech Republic (Act No. 373/2011 Coll. 2011), Ireland (Disability Act 2005) and Lithuania (Civil Code of the Republic of Lithuania 2000, Art. 6.725)]. In countries where the process of genetic testing is not regulated by specific laws or provisions, as long as such tests are considered to be health services, relevant legislation may apply [for example in Denmark (Danish Act on Health 2016), and Slovenia (Health Care and Insurance Act 1992)], as well as regulation related to patient rights (European Commission 2002) and healthcare professionals’ duties (available in most countries).

The regulation of the framework within which the tests are provided, and, more specifically, issues related to appropriate medical supervision, genetic counselling and adequate informed consent process, may also be influenced by international documents such as the Convention on Human Rights and Biomedicine (Oviedo Convention), issued by the Council of Europe and its Additional Protocol on Genetic Testing for Health Purposes, which is the first international binding document (upon ratification) specifically addressing genetic testing (Lwoff 2009). The Oviedo Convention, which aims at protecting human dignity and identity and sets out fundamental principles applicable to daily medical practice (Council of Europe 1997), restricts (as it will be elaborated below) the use of predictive, carrier and predisposition genetic tests to health purposes or scientific research linked to health purposes and it mandates genetic counselling for these tests. The Additional Protocol on Genetic Testing for Health Purposes (Council of Europe 2008) touches upon issues of clinical utility, medical supervision, genetic counselling and informed consent in the context of genetic testing. It has been both signed and ratified by only four members of the Council of Europe, namely Moldova, Montenegro, Norway and Slovenia (Council of Europe 2017a). In addition, in October 2017, the Additional Protocol was also signed by the Czech Republic, which is determined to ratify it (Council of Europe 2017b). However, until the Additional Protocol has been ratified by a fifth country, it cannot come into force, since, this requires at least five ratifications (Council of Europe 2008).

The different above-mentioned regulatory approaches have resulted in a fragmented landscape when it comes to the regulation of DTC genetic testing. In this article, we provide an overview of the wide spectrum of national laws regulating genetic tests which could potentially impact DTC genetic testing in 26 European countries. Particular emphasis is placed on provisions relating to three specific issues: medical supervision, genetic counselling and informed consent. Furthermore, the desirability of adopting a harmonized regulatory framework across Europe with regard to these issues is discussed in the light of the recent adoption of the EU Regulation on IVD medical devices and the debate that preceded its adoption.

## Methods

Experts in health law and/or regulation of genetic testing from countries where the Regulation on IVD medical devices will apply (i.e. EU Member States, the countries of the European Free Trade Association and Turkey) were contacted by LK, HCH and PB and invited to contribute to this study. Experts were asked to answer questions regarding national laws pertinent to the regulatory control of DTC genetic testing services in the country where they have an expertise on the legal framework. Particular emphasis was placed on whether medical supervision and genetic counselling are mandatory and whether there are specific requirements for informed consent in the context of genetic testing.

Each collaborator’s answers constituted what we refer to, as a ‘national report’ which was returned to LK, who then analysed, summarized and grouped the legislations into categories based on similarities. Experts were then re-contacted in order for them to clarify any details regarding their initial answers, ensure the correct interpretation of their reports and the accuracy of the final text. All collaborators are co-authors of this study. One exception to this approach is the information included regarding Portugal; this information was largely based on the description of the Portuguese legal context in the article ‘Legislation on direct-to-consumer genetic testing in seven European countries’ (Borry et al. 2012). The author who contributed to that article is acknowledged herein.

## Results

The results presented below focus on information regarding the way medical supervision, genetic counselling and informed consent in the context of genetic testing are regulated in 26 different European countries. These countries are Austria, Belgium, Cyprus, the Czech Republic, Denmark, Estonia, Finland, France, Germany, Greece, Hungary, Ireland, Italy, Latvia, Lithuania, Luxembourg, the Netherlands, Norway, Poland, Portugal, Romania, Slovakia, Slovenia, Spain, Sweden and the United Kingdom (UK).

### Is medical supervision mandatory in the context of genetic testing?

Medical supervision in the context of health-related genetic testing has been assigned particular importance, as it aims to assist patients in making informed healthcare choices and ensure that they have access to high-quality tests that are appropriate for them. In this regard, Article 7 of the Additional Protocol on genetic testing for health purposes prescribes that ‘A genetic test for health purposes may only be performed under individualized medical supervision’ (Council of Europe 2008). Of the countries included in our study, only *Slovenia, Norway* and recently the *Czech Republic* have agreed to be bound by it. However, to date, none of these countries have put into force provisions rendering health-related genetic testing performed without medical supervision illegal.

Ten of the countries included in our study prescribe mandatory medical supervision and restrictions in the way some genetic tests are performed (Table 1). These countries are *Austria, France, Hungary, Italy, Germany, Austria, Lithuania, the Netherlands, Portugal and Spain*. Despite not having signed or ratified the Additional Protocol, these countries are, therefore, in line with Article 7.

**Table 1:** Countries requiring medical supervision

Country	Types of tests requiring medical supervision	Qualifications of medical professionals	Statute
Austria	GT providing information on: • Carrier status, • A risk of a disease, • An existing disease • A course of an illness or of a therapy on man	A medical specialist trained in human genetics or an attending or a diagnosing medical specialist	Austrian Gene Technology Act Art.68 (1)
France	GT for health purposes	A geneticist or a non-geneticist familiar with the medical situation of the patient who works in close relationship with a center of reference	(1) Civil Code art. 16 (1) (2) Arrété de bonnes pratiques juin 2013 art. 2.2
Germany	GT for health purposes	• Predictive genetic examinations: restricted to physicians specialized in human genetics or other specialized physicians qualified to conduct them in their specialist area of practice • Diagnostic genetic examinations: any physician licensed to practice medicine	Genetic Diagnosis Act
Hungary	GT conducted for: • Prophylactic • Diagnostic • Therapeutic • Rehabilitation purposes • Research purposes based on health reasons	Healthcare providers holding the operating license stipulated in the Minister of Health, Social and Family Affairs Decree on the professional minimum conditions necessary for health care provision	Act No XXI of 2008 on the protection of human genetic data and the regulation of human genetic studies, research and biobanks art. 12
Italy	• Pre-symptomatic GT • Susceptibility GT	Not specified	General Authorisation No. 8/2014 for the Processing of Genetic Data
Lithuania	GT for health purposes	Physicians trained in human genetics	Order No. V-220 issued on April 24, 2003 by Minister of Health
The Netherlands	High-risk diagnostic medical devices (including tests for the diagnosis of hereditary diseases, and predictive genetic tests)	Doctor or a pharmacist	Decree on In-Vitro Diagnostic Devices
Slovenia	GT for health purposes	Clinical Geneticist	Act ratifying the Additional Protocol to the Convention on Human Rights and Biomedicine concerning Genetic Testing for Health Purposes, Art. 7
Portugal	GT for: • The detection of heterozygosity status of recessive diseases • Presymptomatic diagnosis of monogenic diseases • Genetic susceptibility tests in healthy individuals	Clinical geneticist	Law 12/2005 of 26 January 2005, Art. 9.2
Spain	GT for health purposes	Performed by qualified personnel and carried in certified centers)	Act 14/2007, art 56

More specifically, in *France*, tests can only be performed for healthcare purposes, with a medical prescription and realized by an authorized laboratory (Code Civil 2006, article 16-10 and 16-11; Code de la santé piblicque 1953, article R. 1131-9 and R. 1131-14). The professional prescribing genetic tests can be either a geneticist or a non-geneticist, as long as he/she is familiar with the medical situation of the patient and he/she works in close relationship with a reference centre (Arrété de Bonnes Pratiques 2013, article 2.2). What distinguishes France from other countries with restrictive regulatory frameworks when it comes to genetic testing, is that France also introduces penalization of the users (i.e. consumers ordering a test outside the clinical setting). More specifically, the infringement of this provision is punishable under the criminal code by a fine of 3.750 euro (Borry et al. 2012).

In *Hungary*, genetic tests may only be performed by healthcare providers^3^ (Genetic Act 2008, article 12) and only for specific purposes based on health reasons, namely, for prophylactic, diagnostic, therapeutic, rehabilitation or research purposes (Genetic Act 2008, article 13). Similarly, in *Italy*, pre-symptomatic and susceptibility tests are only permitted for healthcare purposes and healthcare-related research purposes (Italian General Authorization for the Processing of Genetic Data 2014). These tests depend on medical prescription and *ex ante* supervision should, therefore, take place.

In *Germany*, the Genetic Diagnosis Act (Gendiagnostikgesetz 2009) prescribes that, whenever a genetic examination is conducted for medical purposes, supervision by a qualified physician is mandatory. In this regard, a distinction is made between the qualifications required by physicians conducting diagnostic genetic examinations and those required by physicians conducting predictive genetic examinations (sec. 7 para. 1). Namely, diagnostic genetic examinations may be performed by any physician licensed to practice medicine, whereas predictive genetic examinations may only be performed by physicians specialized in human genetics or by other specialized physicians qualified during their medical training to perform them in their specialist area of practice. The Genetic Diagnosis Act also stipulates that genetic examinations for the purpose of clarifying a family relationship can only be carried out by physicians or qualified non-medical experts (sec. 17 para. 4).

In the *Netherlands*, according to the Decree on In Vitro Diagnostic Devices (Besluit in-vitro diagnostica 2001), ‘high-risk diagnostic medical devices’ including tests for the diagnosis of hereditary diseases, and predictive genetic tests may not be supplied directly to the user without the intervention of a doctor or pharmacist.

In addition, *Austria* (Gentechnikgesetz 1994, article 68.1)*, Lithuania* (Order No. V-220 2003)*, Portugal* (Lei no 12/2005 2005) *and Spain* (Boletín Oficial del Estado No159 2007, articl 56) confine the provision of most health-related genetic tests in designated facilities and require supervision by a healthcare professional.

In countries without specific legislation on genetic testing, the question of whether DTC genetic testing could be defined as a health service or as an informational/recreational/educational activity has a rather important impact on the way such tests may be regulated across different European countries.

For example, in *Belgium*, sanctions could be imposed on an illegal practice of medicine (Law on the Practice of Health Care Professions 1967, article 3). Hence, if offering a DTC genetic test would be considered to constitute practice of medicine, the Law on Patient Rights would apply and a physician should be involved in the provision of the tests. However, many DTC companies write in their ‘terms of services’ that they are not practicing medicine, and that their tests should not be considered medical information, but only serve ‘informational purposes’.

Similarly, in *Denmark* the application of the Act on Authorisation of Healthcare Professionals is contingent to whether DTC genetic tests are considered to be health services or not. According to this Act, it is generally legal for laypersons without professional authorisation, to treat patients, provided they do not expose the patient to danger (Danish Authorization Act 2016). However, certain kind of treatments and procedures, outlined in section 74 of the Act may only be carried out by (or under the supervision of) a medical doctor. If laypersons’ treatment is not compliant with these rules, criminal sanctions may be imposed. The procedures normally involved in DTC genetic testing (mouth swab) are not listed as one of the treatments or procedures exclusively assigned to medical doctors. Consequently, it is only in situations where the test exposes the patient to danger, that sanctions can be employed.

### Is genetic counselling mandatory in the context of genetic testing?

Similar to the goals of medical supervision, but with a more specific process and aim, genetic counselling is a communication process aiming to support patients in taking informed healthcare decisions, after understanding the benefits, limitations and implications of a genetic test for themselves and their family and being informed about available healthcare options.

Currently, there are 16 countries requiring genetic counselling for some types of genetic tests: *Austria, Cyprus, the Czech Republic, Estonia, Finland, France, Germany, Greece, Hungary, Italy, Latvia, Lithuania, Norway, Slovakia, Slovenia and Spain* (Table 2).

**Table 2:** Countries requiring genetic counseling

Country	Types of genetic tests requiring genetic counseling	Qualifications of medical professional	Statute
Austria	GT for: • Determination of a manifested disease, which is based on a germ line mutation • Determination of predisposition for future onset disease or carrier status for which a prophylaxis or therapy is possible • Determination of predisposition for future onset disease or carrier status for which a prophylaxis or therapy is not possible • Prenatal diagnosis	Medical specialist with an education in human genetics/medical genetics or a medical specialist competent for the respective specialty.	Austrian Gene Technology Act, Art 69
Cyprus	• Prediction of disease • Carrier GT • GT detecting predisposition or susceptibility to a disease	Not specified	Law 31 (III)/2001 art. 12
Czech Republic	GT for health purposes	Not specified	Act no 373/2011 Coll. On specific Health Care Services
Estonia	GT for health purposes	Not specified	Oviedo Convention
Finland	GT for health purposes	Not specified	Oviedo Convention
France	GT • Giving, confirming or refuting the diagnosis of a genetic disease • Detecting characteristics of one or more genes which may be the cause of developing a disease	Geneticist or genetic counselor	Arrêté de bonnes pratiques Juin 2013
Germany	• Predictive GT, prenatal GT, fetal aneuploidy risk assessment by non-invasive measures (obligatory pre- and post-test counseling) • Diagnostic GT (post-test counseling should be offered. In case, the test result reveals an untreatable disease, post-test counseling is obligatory) performed)	Physician specialized in human genetics or additionally qualified in medical genetics or physician qualified in genetic counselling in her or his area of practice either through post-qualification professional training (compulsory since February 2012) or during specialty training (currently in preparation).	Genetic Diagnosis Act
Greece	• GT predictive of disease • Carrier GT • GT detecting predisposition or susceptibility to a disease	Not specified	Law 2619/1998 art. 12
Hungary	Genetic testing and screening	Not specified	Parliamentary Act No XXI of 2008 on the protection of human genetic data, on the human genetic studies on research and on the operation of the biobanks. Amended by the Act no CLXXVI of 2011 and by the CXXVII of 2013.
Italy	GT for: • Health purposes • Family reunion purposes	Physician or specialist in genetic medicine	General Authorisation No. 8/2014 for the Processing of Genetic Data by the Data Protection Authority, point 5.1
Latvia	GT for health purposes	Not specified	Oviedo Convention
Lithuania	GT for health purposes	Not specified	Oviedo Convention
Norway	• Presymptomatic GT • Predictive GT • Carrier GT	Not specified, but according to the preparatory works (Ot.prp. No. 62 (2002–2002)) GT should preferably be provided by specially trained health personnel. If other kind of health personnel carry out GT, the general rule of professional responsibility and diligent care apply.	Act of 5 December 2003 No 100 relating to the application of biotechnology in human medicine
Slovakia	GT for health purposes	Not specified	Oviedo Convention
Slovenia	• Predictive GT • Carrier GT • Predisposition/Susceptibility GT • Prenatal GT	Clinical geneticist	Act Ratifying the Convention for the Protection of Human Rights and Dignity of the Human Being with regard to the Application of Biology and Medicine: Convention on Human Rights and Biomedicine, and of the Additional Protocol to the Convention for the Protection of Human Rights and Dignity of the Human Being with regard to the Application of Biology and Medicine, on the Prohibition of Cloning Human Beings. UL RS 17/98., Art. 12
Spain	GT for any health purposes	By qualified personnel and carried in certified centers	Act 14/2007, Art 55 and 56

The relevant legislation of some of these countries have been influenced by the Convention for the Protection of Human Rights and Dignity of the Human Being with regard to the Application of Biology and Medicine: Convention on Human Rights and Biomedicine (hereinafter Oviedo Convention) and, more specifically, Article 12 on ‘Predictive genetic tests’. Article 12 of the Oviedo Convention prescribes that

tests which are predictive of genetic diseases or which serve either to identify the subject as a carrier of a gene responsible for a disease or to detect a genetic predisposition or susceptibility to a disease may be performed only for health purposes or for scientific research linked to health purposes, and subject to appropriate genetic counselling (Council of Europe 1997).

Of the 26 countries included in our study, the following 16 have both signed and ratified the Oviedo Convention: *Cyprus, the Czech Republic, Denmark, Estonia, Finland, France, Greece, Hungary, Latvia, Lithuania, Norway, Portugal, Romania, Slovakia, Slovenia and Spain.* The ratification of the Convention creates the obligation for those countries to introduce implementing legislation in their national law, bringing it to conformity with the principles of the Convention (Andorno 2005).

In *France* genetic counselling is mandatory and can be performed either by a geneticist or a genetic counsellor (Arrété de Bonnes Pratiques 2013). Similarly, in *Spain* (Boletín Oficial del Estado No159 2007) and the *Czech Republic* (Act No. 373/2011 Coll. 2011), when it comes to health-related genetic testing, the patient must be guaranteed appropriate genetic counselling. In the case of Spain, the qualified professional who carries out or coordinates the genetic counselling must provide adequate information in relation to both the consequences of the resulting genetic diagnosis, as well as the possible alternatives available to the patient.

Under the *Hungarian* law (Parliamentary Act No XXI 2013), genetic counselling is also mandatory and is defined as a consultation procedure during which a person authorised by special legislation provides information on the advantages or risks of human genetic studies, explores possible consequences of the results of genetic studies and helps to understand the nature of the illness.

In *Greece* (Law 2619/1998 1998, article 12) and *Cyprus* (Law 31 (III)/2001 article 12), appropriate genetic counselling is mandatory for predictive, carrier and predisposition genetic tests. In *Norway* (Bioteknologiloven 2003) genetic counselling is compulsory when it comes to presymptomatic, predictive and carrier test. On the same line, *Slovenia* (UL RS 17/98 1998, article 12), where national legislation regarding genetic testing is currently being developed, all presymptomatic, predictive and prenatal genetic tests are performed with pre- and post-test genetic counselling by a clinical geneticist.

Other countries, such as *Denmark,* 4 *Estonia, Finland, Latvia, Lithuania, Slovakia and Romania*, despite having signed and ratified the Oviedo Convention, do not stipulate explicitly in their national legislation that genetic counselling should be mandatory for the provision of health-related genetic testing. Nevertheless, in some of these countries (for instance, in *Estonia, Finland, Latvia, Lithuania* and *Slovakia*) ratified international treaties are directly applicable. Therefore, the requirements regarding genetic counselling imposed by Article 12 of the Oviedo Convention are applicable even without being incorporated into national law (Eurogentest 2002).

Countries that have only signed but not ratified the Oviedo Convention (so although they acknowledge the principles underlined by the Convention, they have no obligation to introduce its principles into their national law) are *Italy, Luxembourg, the Netherlands,* 5 *Poland and Sweden.*

In *Italy,* the Parliament has authorized the ratification and has incorporated the Convention in the domestic system, but has not yet deposited the instruments of ratification. In this regard, in Italy, genetic counselling is mandatory. This should be the case not only for health-related genetic tests, but also for genetic tests informing on family relationships. This is because based on the General Authorisation No. 8/2014 for the Processing of Genetic Data by the Data Protection Authority: ‘With regard to processing operations carried out via genetic tests for healthcare and/or family reunion purposes, genetic counselling shall be provided to data subjects both before and after performing the tests’ (Italian General Authorization for the Processing of Genetic Data 2014).

*Austria, Belgium, Germany, Ireland* and the *UK* have neither signed nor ratified the Oviedo Convention (Council of Europe 2017a). Despite this fact, Austrian and German laws provide a detailed framework for genetic counselling in the context of genetic testing.

In *Austria,* the Austrian Gene Technology Act (hereinafter GTG) makes the provision of genetic counselling mandatory for certain categories of tests and provides detailed and strict conditions for its provision (Gentechnikgesetz 1994, art. 69). More specifically, certain types of genetic tests^6^ may only be performed after the person taking the test has been informed about the genetic test’s nature, consequences and significance. This consultation should be non-directive and performed by a medical specialist with an education in human genetics/medical genetics or a medical specialist competent for the respective specialty. The test may only be performed if the patient has agreed to the genetic test as a result of a free consent based on that knowledge. Post-test genetic counselling has to include a comprehensive discussion of all test results and medical facts as well as possible medical, social and psychological consequences. When it comes to genetic testing for predisposition to a hereditary disease with serious physical, mental or social consequences, an additional nonmedical counselling by a psychologist or a psychotherapist or by a social worker should be recommended to the patient in written form.

In *Germany,* according to the Genetic Diagnosis Act (Gendiagnostikgesetz 2009) predictive genetic testing (sec. 10 para. 2), foetal aneuploidy risk assessment by non-invasive methods and any prenatal genetic examination (sec. 15 para. 3) require pre- and post-genetic counselling. For diagnostic genetic examinations, post-test counselling should merely be offered (sec. 10 para. 1). An obligation to perform post-test genetic counselling exists only for test results referring to a condition for which no treatment is currently available. Genetic counselling may only be performed by physicians who are specialists in human genetics, who are additionally qualified in medical genetics or who are qualified in genetic counselling in their area of practice through post-qualification professional training (sec. 7 para. 3).

### Are there specific requirements for informed consent that may apply in the context of genetic testing?

Fourteen of the countries examined include specific provisions regarding informed consent in the context of genetic testing (Table 3). These countries are *Austria, the Czech Republic, France, Germany, Hungary, Ireland, Italy, Norway, Portugal, Slovakia, Slovenia, Spain, Sweden* and the *UK.*

**Table 3:** Countries requiring written informed consent specifically for genetic testing

Country	Types of tests	Statute
Austria	GT for • Determination of a manifested disease, which is based on a germ line mutation • Determination of predisposition for future onset disease or carrier status for which a Prophylaxis or therapy is possible • Determination of predisposition for future onset disease or carrier status for which a prophylaxis or therapy is not possible • Prenatal diagnosis	Austrian Gene Technology Act, Art 69
Czech Republic	All GT	Act No 373/2011 Coll. On Specific Health Care Services
France	• Genetic tests provided for medical purposes including pre-transfer and prenatal tests. • Processing of genetic data for research.	Civil code, Art. 16-10, Public Health Code, Art. L 1131-1, Art. L 2131–4 Act N°78-17 of 6 January 1978 on Information Technology, Data Files and Civil Liberties, Art. 56
Germany	• GT for medical purposes • GT to determine a family relationship	Genetic Diagnosis Act
Hungary	All GT	Parliamentary Act No XXI of 2008 on the protection of human genetic data, on the human genetic studies on research and on the operation of the biobanks. Amended by the Act no CLXXVI of 2011 and by the CXXVII of 2013. Article 6 (4)
Ireland	Informed consent is required for the processing of genetic data	Disability Act 2005, Part 4, Section 41
Italy	• Pre-symptomatic GT • Susceptibility GT	General Authorisation No. 8/2014 for the Processing of Genetic Data
Norway	All types of analyses of human genetic material at both nucleic acid and chromosome level, analyses of genetic products and their function, and examination of organs to obtain information on human genetic constitution	Act of 5 December 2003 No. 100 relating to the application of biotechnology in human medicine
Portugal	GT for: • The detection of the heterozygosity status of recessive diseases, • The presymptomatic diagnosis of monogenic disease • Genetic susceptibility in healthy individuals	Law no 12/2015
Slovakia	All GT	Act No 122/2013 Coll. On Personal Data Protection
Slovenia	Health-related GT	Act ratifying the Additional Protocol to the Convention on Human Rights and Biomedicine concerning Genetic Testing for Health Purposes, UL RS 14/09.
Spain	GT performed in a health context	Act 14/2007, Art 45 d) and 48.1
Sweden	Genetic screening (not individual testing)	The Genetic Integrity Act chapter 3, section 1 (2006:351)
UK	The Act makes it an offence to carry out a genetic test without “qualifying consent” with a small number of exceptions. Consent may be written or oral. According to the HTA’s website: “All companies providing DNA testing kits or DNA testing services must comply with the provisions of the Human Tissue Act 2004 relating to consent and the holding of bodily material with the intent to analyze DNA.”^a^	Human Tissue Act 2004 (s 45 (1)(a)(i)

^a^HTA, ‘Analysis of DNA under the HT Act FAQs’ https://www.hta.gov.uk/faqs/analysis-dna-under-ht-act-faqs accessed 10 October 2016

For example, according to the *German* Genetic Diagnosis Act (sec. 9 para. 2), the physician who intends to undertake a genetic examination is under an obligation to provide prior information to the patient regarding the purpose, type, scope, and significance of the examination and the anticipated meaningfulness of the results (Gendiagnostikgesetz 2009). The information to be given must also cover the potential health risks for the patient associated with obtaining the sample and knowledge of the test results. Moreover, the patient must be informed about his/her right ‘not to know’. The patient is entitled to withdraw his/her consent to the genetic examination and/or to the disclosure of the results and to demand that they be destroyed at any time before they are received. The information must be summarised in a written document before the examination takes place and appropriate time for consideration must be granted to the subject person to decide whether to consent or not (sec. 9 para. 1). An essential precondition of the performance of a genetic examination is express written consent (sec. 8). Non-compliance with this requirement is punishable by imprisonment or a fine (sec. 25). The information must be given orally but can be supplemented by written documents (German Civil Code 1900, sec. 630e para. 2).

In *France* very specific provisions have been adopted to ensure a particular protection of individuals with regard to the prescription and information on results of genetic testing. Prior information must be delivered to the patient by the physician who is prescribing the analysis. This information concerns (1) the characteristics of the sought disease, (2) the means of detecting it and (3) the possibilities of prevention and treatment. The information must be summarised in a written document which could be used to inform the family members. Only the prescribing medical doctor can deliver the information on the results to the patient who has the right not to know. This information must be included in the medical record.

In *Spain*, the conditions for informed consent are regulated for all genetic tests performed in a health context. According to Act 14/2007 (Boletín Oficial del Estado No159 2007, article 45 d and 48.1), the patient must receive written information regarding: (1) the purpose of the genetic analysis; (2) the place where the analysis shall take place and the way in which the biological sample will be treated at the end of the analysis, whether it is the disassociation of the identifying data from the sample, its destruction or other treatments; (3) the persons who will have access to the results of the analysis when samples will not undergo a process of disassociation or anonymisation; (4) a warning about the possibility of unexpected findings and the possible implications for him or her, as well as the patient’s right not to know; (5) a warning about potential implications of this information for his or her family members, and their interest, where appropriate, in having that information conveyed to them; (6) an agreement to provide genetic counselling, once the results of the analysis are obtained and evaluated.

In *Hungary,* the person undertaking a genetic test shall be entitled to learn about the results of the human genetic examination in a consultation that is specifically tailored to his/her needs (Parliamentary Act No XXI 2013). During the consultation the person concerned shall be assisted in the long-term processing of the possible consequences of the result and the choice of optimum treatment opportunities.

*The Czech Republic* (Act No. 373/2011 Coll. 2011) and *Slovenia* (UL RS 17/98 1998) mandate that written informed consent is a prerequisite for all genetic tests. On the same line, *Ireland* (Disability Act 2005) and *Slovakia* (Act No. 373/2011 Coll. 2011) require informed consent for the ‘processing’ of genetic data.

Other countries, such as *Austria, Portugal, and Norway* require written informed consent only for specific categories of genetic tests. More specifically, in *Austria,* genetic tests determining a manifested disease based on a germ line mutation, genetic tests determining predisposition to a disease (especially future onset diseases or the determination of carrier status), as well as genetic tests for prenatal diagnosis, are only allowed to be performed upon written consent of the person to be tested within the context of the genetic counselling as described above. The informed consent form should indicate that the patient has been informed in advance about the genetic test’s nature, consequences and significance. The information should be provided by a medical specialist with an education in human genetics/medical genetics or a medical specialist competent for the respective specialty (Gentechnikgesetz 1994, article 69).

In *Portugal* (Law n 12/2005 2005, article 9.2)*,* 7 detecting heterozygosity status of recessive diseases, diagnosing monogenic diseases and testing for genetic susceptibility in healthy individuals may only be carried out upon explicit written informed consent of the person undertaking the examination (Borry et al. 2012). In *Norway*, written consent shall be obtained from the person concerned, before presymptomatic, predictive and carrier genetic tests are undertaken (The Patients’ Rights Act 1999, d sect. 5-1, second paragraph, litra b). Before such testing is carried out, the Ministry shall give separate authorization for each disease or predisposition to a disease that is to be tested (The Patients’ Rights Act 1999, sect. 5-3). In *Sweden,* written consent is required for genetic tests that are carried out as part of a medical screening (Lag (2006:351) 2006, section 1) (but not necessarily if it is a service provided to one person based on individual needs).

In countries where specific informed consent rules regarding genetic testing are not in place, general laws may apply in this context, many of them related to patient’s rights and doctor’s obligations.

For example, in *Denmark,* if a medical doctor or another licensed healthcare professional is involved in (DTC) genetic testing, the general obligation to provide due care applies and so do the provisions in the Danish Act on Health regarding patients’ right to information, consent and privacy. Although these rules are not specifically targeting genetic testing, they have an impact on, for example, the right to information both before and after the test, the right not to know as well as protection of genetic information and regulation of access to and disclosure of information both within the healthcare services and to third parties. The Act on Health also contains special provisions regarding young patients’ right to self-determination and proxy consent in regard to children and non-autonomous adults, which are of importance for genetic testing.

On the same line, according to the *Dutch* ‘Medical Treatment Contracts Act’, (laying down the rights and obligations of care providers and patients, as well as, in certain situations, customers) healthcare providers should provide information about the indication, the proposed treatment, alternatives, prognoses have, risks and possible side effects before starting a medical intervention. Furthermore, a healthcare worker always needs informed consent of the patient in order to perform any test, including a genetic test. The patient can also decide that he/she wishes to be spared certain information or the results.

## Discussion

### To what extent do these laws affect DTC genetic testing?

Based on the above information, it is evident that European countries follow different approaches when it comes to regulation of genetic testing and impose various levels of restrictions. On the one end of the spectrum is *France,* which essentially bans DTC genetic testing, by limiting the use of genetic testing to specific health-related tests, mandating the involvement of healthcare professionals and penalizing users of tests that do not fulfil these conditions. Similarly, *Germany* has legislation that has been considered by some as targeting DTC genetic testing and limiting the access German consumers may have to this type of testing (EASAC and FEAM Working Group 2012; C. Wright 2009). On the other end are many countries that do not provide any specific legislation on genetic testing (such as *Luxembourg, Poland* and *Romania*), and, as a result, the only restrictions in place are those based on more general laws, usually regarding healthcare services and patients’ rights.

It is worth noticing that the aspects of genetic testing that this article particularly addresses, namely medical supervision, genetic counselling and informed consent, are closely connected and often overlap in practice. For example, it is often the case that informed consent is obtained from the patient during a genetic consultation performed by a healthcare professional who may have also prescribed the test. When it comes to health-related genetic testing, medical supervision, genetic counselling and informed consent have some fundamentally similar goals, namely to guide users to make decisions about their health based on genetic tests that are appropriate for them, after understanding their benefits, limitations and possible implications. The close connection of these notions can be noticed in some national laws that do not address these aspects separately. In many jurisdictions (for example in *Sweden* and *Ireland*), despite genetic counselling not being specifically addressed, its necessity may be implied by provisions relevant to informed consent. This is because it may be argued that, in the context of genetic testing, unless genetic counselling takes place, consent cannot really be informed.

Most of the above-mentioned laws apply to genetic testing offered within the conventional healthcare system, where laboratories and clinical services are subject to professional bodies and internal controls (C. F. Wright et al. 2011). Applying the same laws in the commercial sector, outside of a clinic or hospital may be more complicated.

While in some cases (such as in the case of *Germany, France* (Abbasi and Rial-Sebbag 2012) and *Spain*) even if the laws do not explicitly refer to the commercial context, it seems to be evident that the law restricts the provision of DTC genetic tests. In *Hungary,* the Genetic Act of 2008 has a broad scope, originally tailored to the public healthcare system, but it also applies to private companies operating in Hungary in that it explicitly regulates the type of tests that are allowed. In other countries, however, it is not clear whether the law only regulates the genetic testing process within the clinic or whether relevant provision could be the basis for banning or limiting DTC companies from marketing their products in specific European countries (Grimaldi et al. 2011).

In the *UK,* the distinction between the regulation of genetic testing within the clinical setting and the provision of genetic testing DTC seems to be clearer. While in the clinical setting there is medical supervision and genetic counselling available, DTC genetic testing (that often does not fulfil those requirements) is currently not prohibited in the country. A number of companies are selling DTC services to UK consumers and 23andMe is currently selling its kits both through its website and through the pharmaceutical chain Superdrug (GeneWatch UK 2015). A similar approach also applies in *Estonia* where various nutrigenetic and health-related diagnostic^8^ tests are sold via pharmacy chains. However, all prenatal tests for the diagnosis and treatment of diseases listed in International Statistical Classification of Diseases and Related Health Problems Tenth Revision (ICD-10) are carried out only in clinical centres as health service according to the Health Services Organisation Act.

Another crucial point to be taken into account when considering whether some laws apply to DTC genetic tests is whether these tests are considered to be health services or not. DTC genetic testing often blurs the lines between medical devices and consumer products. In some cases, it is unclear whether providing these kinds of services may constitute practice of medicine or not. The answer to this question may determine whether several national laws, such as the Health and Medical Services Act and the Genetic Integrity Act that regulates uses of certain biotechnology for medical purposes in *Sweden*, the Medical Treatment Law in *Latvia* and the Act on Authorization of Healthcare Professionals in *Denmark*, as well as various national laws related to patients’ rights, apply in this context.

A straightforward answer is not evident; this is because there is no uniform definition across European countries of what constitutes ‘practice of medicine’. This matter is usually left to national medical licensing authorities to decide (Marietta and Mcguire 2009). In addition, the distinction between health-related and non-health related tests is not always clear. Health-related tests have been given various definitions. For example, Goddard et al. define them as tests that aim ‘to predict risk of disease, screen for disease, direct clinical management, identify carriers, or establish prenatal diagnoses, clinical diagnoses, or prognoses in individual people or families’ (Goddard et al. 2008). There are several types of genetic tests that, despite not falling in the above mentioned categories, can still have an (indirect or direct) impact on health, such as nutrigenomic tests (Grimaldi et al. 2011). Moreover, many of the ‘packages’ of tests currently available provide medical, genealogical and recreational information at the same time, challenging the distinction between medical and lifestyle devices (Lucivero and Prainsack 2015; Prainsack et al. 2008). At the same time, this matter is further complicated by the rhetoric adopted by many DTC genetic testing companies, which often underplays the health implications of their services and stresses the ‘informational’ and ‘fun’ aspects of their services (Kalokairinou et al. 2017).

A further challenge for the effective regulation of DTC genetic testing is the fact that these tests are mostly sold through the internet and hence where different jurisdictions begin or end is difficult to clearly delineate. DTC genetic testing companies may be based anywhere in the world, and, indeed, most of them are based outside Europe. As a result, even if a test is sent to a specific European country, the regulation of this service may (or may not) still fall outside the national jurisdiction. For example, in *Norway,* the Act of 5 December 2003 No 100, relating to the application of biotechnology in human medicine stipulates in regard to the territorial scope that it ‘applies within the realm’. As a result, providers operating abroad would clearly fall outside the territorial scope of the act, even if the enterprise is directly targeting the Norwegian market. Moreover, even if these services fall within the scope of national laws, their enforcement may be rather challenging. This is because, as Hauskeller has claimed, ‘A globally acting, internet based industry cannot be forced to comply with laws or regulations that are binding only country by country’ (Hauskeller 2011).

It should be also noted that besides laws related to medical supervision, genetic counselling and informed consent, DTC genetic testing companies may encounter additional regulatory barriers when selling their products in some European countries. For example, in *the Netherlands*, the Dutch Act on Population Screening (Wet op het bevolkingsonderzoek 1992) may require such companies to obtain a license before offering their products to the Dutch population. According to this Act, a population screening is a medical examination carried out in response to an offer made to the entire population or to a section thereof, in order to detect certain diseases or risk indicators. Screenings that use ionizing radiation, for (risk-indicators for) cancer and untreatable diseases require a license by the Minister of Health, Welfare and Sports due to their potential health risks.^9^ Commercial companies offering such genetic tests DTC may fall within the scope of the Act, since ‘offer’ in this context may not be limited to actively inviting individuals, but also the passive ‘seduction’ of people via advertisements.

Finally, it should be underlined that the practical impact of such laws on DTC genetic companies is difficult to be precisely estimated. Currently, some companies, such as 23andMe only market their products to specific European countries, possibly in order to avoid legal repercussions in countries with more restrictive regulatory framework. However, while in the US, there have been several legal actions against DTC genetic testing companies both by governmental bodies, such as the Food and Drug Administration (FDA 2013) and the Federal Trade Commission (US Federal Trade Commission 2014), as well as by consumers (Munro 2013), we are not aware of similar legal actions having been taken in the countries covered by our study.

### A missed opportunity for a harmonized regulatory framework?

The current fragmented regulatory framework across Europe regarding medical supervision, genetic counselling and informed consent as well as the uncertainties regarding the scope of application of national laws brings up the question of whether a harmonized European regulation would be desirable.

This question became particularly topical during the revision process of the Medical Devices Directives (European Commission 2017). This process led to the adoption, on 5th April 2017, of the Regulation on IVD medical devices, which will replace the IVD Directive. During the ordinary legislative procedure (European Parliament 2015), the European Parliament issued a proposal suggesting that this instrument should also regulate aspects related to medical supervision, genetic counselling and informed consent (Committee on the Environment Public Health and Food Safety 2013). More specifically, the European Parliament’s proposal suggested, among others, that all genetic tests with a direct or indirect medical purpose should be classified as prescription only devices, and that their DTC advertising should be forbidden. In addition, it was proposed that genetic counselling should be mandatory for predictive, prenatal and diagnostic genetic tests. The counselling for this kind of tests, according to the proposal, should be intelligible and cover medical, ethical, social, psychological and legal aspects, while informed consent should be written (Committee on the Environment Public Health and Food Safety 2013; Kalokairinou et al. 2015). The adoption of these amendments would have effectively signalled a ban on most types of DTC genetic testing. Such a ban would have been uniformly implemented across Europe, since Regulations, as opposed to Directives are directly binding for Member States, without the need of being transposed into the national legislation (European Union 2017).

The proposal received strong criticism regarding these amendments. The criticism was mostly focused on whether the EU has the competence to enact such amendments and whether the amendments would actually be beneficial for clinical practice. A legal opinion issued by members of the Alliance of European Life Science Law Firms (hereinafter the Alliance Opinion) (Alliance of European Life Sciences Law Firms 2014) stated that the amendment on informed consent and genetic counselling of the proposal would infringe the principles of proportionality and subsidiarity, established by the Treaty on the Functioning of the European Union. According to these principles, the EU may only take legislative action in cases where the objectives of the legislation may be achieved more effectively at an EU level rather than at a Member State level, by reason of the scale or effects of its proposed action (Alliance of European Life Sciences Law Firms 2014; European Union 2012). According to the Alliance Opinion, there is no evidence that the amendments suggested by the Parliaments’ proposal reflected these principles. These amendments were perceived by many as an attempt to regulate medical practice through regulation on devices (Eurogentest 2014), since the aim of the Regulation on IVD medical devices is the same as this of the Directive: to enhance the smooth functioning of the internal market and to prevent IVD devices that are not safe and efficient from entering the market.

Furthermore, several stakeholders pointed out the potential negative results of imposing uniform strict criteria in clinical practice across Europe. In this regard, it was mentioned that potential adoption of such legislation ‘will mark an unprecedented interference by the European Union in clinical practice’(Eurogentest 2014) and the European Society of Human Genetics considered them to be ‘unworkable in in the daily practice of medicine’. This is largely because, as also indicated by our results, clinical practice is organized in different ways across Europe. It was considered that imposing detailed arrangements for every clinic would

restrict legitimate, ethically acceptable genetic testing activities such as newborn screening and fetal sex determination, and they infringe on accepted and acceptable clinical practice when they should be regulating IVDs, effectively high-jacking a sound and important directive to interfere with carefully regulated clinical practice and infringing on patients’ autonomy (Eurogentest 2014).

Amid those reactions, the original amendments proposed by the European Parliament regarding medical supervision, genetic counselling and informed consent where not included in the final document of the Regulation on IVD medical devices. The text voted by European Parliament mentioned in the recitals:

It appears that divergent national rules regarding the provision of information and counselling in relation to genetic testing may only have an impact on the smooth functioning of the internal market to a limited extent. Therefore, it is appropriate to lay down only limited requirements in this regard in the present regulation, having regard to the need to ensure constant respect of the principles of proportionality and subsidiarity (European Parliament and the Council of the EU 2016).

In this text, the proposed amendment according to which genetic tests should be classified as prescription-only medical devices is omitted. When it comes to informed consent, the newly adopted Article 4a on ‘Genetic information, counselling and informed consent’ stipulates that genetic tests performed in context of healthcare and for the medical purpose of diagnostics, improvement of treatments, predictive or prenatal testing, the individual undergoing testing should receive relevant information regarding the nature, the significance and the implication of the test. Furthermore, the same article stipulates that Member States shall ensure appropriate access to genetic counselling for tests that provide information on the genetic predisposition for medical conditions and/or diseases perceived to be untreatable based on the state of science and technology (European Parliament and the Council of the EU 2016). The Regulation also explicitly mentions that Member States should be free to adopt or maintain in their national legislation more protective provisions regarding informed consent and genetic counselling and national laws classifying specific IVD devices as requiring a medical prescription shall not be affected.

The approach taken in the final text of the Regulation seems to be rather pragmatic: the Regulation focuses on its main aim which is the safety and performance of IVD devices. At the same time, the Regulation places more emphasis on genetic counselling and informed consent compared to the Directive, while leaving Member States leeway to adapt those requirements in their clinical practice. While aspects related to genetic tests as products, such as their clinical validity, will be uniformly regulated by the new Regulation on IVD medical devices in order to enhance the smooth functioning of the internal market, medical supervision, genetic counselling and informed consent are aspects closely related to the clinical practice of each Member State.

A harmonized regulatory approach across Europe on medical supervision, genetic counselling and informed consent for genetic testing may contribute to a more effective oversight of DTC genetic testing services and potentially minimize some of the risks associated with such services. However, the provisions suggested during the debate on the adoption of the Regulation on IVD medical devices, would have also resulted in essential changes in the clinical practices of Member States, which were considered by many unworkable and have been unwelcomed by several stakeholders.

While, these developments may be perceived by some as a missed opportunity towards a uniform pan-European regulatory framework, imposing strict uniform standards of informed consent, genetic counselling and medical supervision by means of an EU Regulation, seems, impractical and restrictive and beyond the legislative competence of the EU. While it is important to ensure equal level of protection of patient rights across Europe, including availability of genetic counselling and medical supervision, as well as adequate informed consent processes, such standards can be achieved by Member States following the Additional Protocol on genetic testing for health purposes (Council of Europe 2008) and relevant international and European guidelines (European Society of Human Genetics 2010; Organisation for Economic Co-operation and Development 2007). Furthermore, emphasis should be placed in coordinating efforts for the harmonization of genetic testing guidelines across Europe.

## Limitations

This article aims to give a general overview of national legislations addressing genetic testing, but it should not be considered as providing an exhaustive list of applicable legislation. Different interpretations of the laws than the ones provided in this article may be possible. This is because, as discussed in this article, DTC genetic testing is regulated by national laws applying to such products by analogy. The applicability of relevant legislation is further complicated by the fact that DTC genetic testing is provided outside the traditional healthcare system, blurring the lines between medical and recreational products. Furthermore, DTC genetic tests are mostly sold through the internet, raising questions regarding jurisdiction and enforcement.

## Conclusion

We provide herein an overview of 26 European countries’ national legislations addressing the topics of medical supervision, genetic counselling and informed consent in the context of genetic testing. The results clearly demonstrate that there is a large heterogeneity in the way these different countries have chosen to regulate these aspects of genetic testing. Furthermore, we have discussed the potential impact of these legislations on DTC genetic testing; with highly varying levels of restrictions, some countries would appear to (almost) ban DTC genetic testing, while others do not appear to have much regulatory framework for this model of provision. With a steadily increasing number of companies and different tests sold, such an overview provides a rich resource that can be used to further understand the legal landscape in Europe and to potentially identify different legislative tools that may be useful in helping guide DTC genetic testing companies to act responsibly.

## Footnotes

1 France can be considered to have a dual system where some provisions on genetic testing are implemented in the bioethics law and others are implemented in the civil code

2 An unofficial English translation can be retrieved from:
https://www.eshg.org/fileadmin/www.eshg.org/documents/Europe/LegalWS/Germany_GenDG_Law_German_English.pdf [the English translation is for information purposes only and has no legal force].

3 These healthcare professionals should fulfil the material and personal conditions and hold the operating license stipulated in the Minister of Health, Social and Family Affairs Decree on the professional minimum conditions necessary for healthcare provision.

4 Despite the lack of laws mandating genetic counselling, providing such consultations may be considered as part of a general obligation to provide due care (section 17 of the Consolidating Act no. 1356 of 23 October 2016 on authorisation of healthcare professionals and on healthcare services)

5 However, recently the Dutch government decided not to ratify the Oviedo Convention because of Article 18: “The creation of human embryos for research purposes is prohibited.”

6 Tests determining a manifested disease based on a germ line mutation, genetic tests determining predisposition to a disease (especially future onset diseases or the determination of carrier status), regardless whether prevention or therapy is possible or not, as well as tests for prenatal diagnosis.

7 Portugal ratified the Oviedo Convention through Presidential Decree n 01/2001, which means that the Convention has now the force of law in the national territory.

8 It should be noted that various diagnostic test-kits are not at all genetic tests but antibody detecting kits.

9 Such license may be granted when the population screening is scientifically sound, in accordance with the professional medical practice standard (informed consent, genetic counseling) and the expected benefits offset the risks
